# The Role of Co-occurring Emotions and Personality Traits in Anger Expression

**DOI:** 10.3389/fpsyg.2018.00123

**Published:** 2018-02-09

**Authors:** Aire Mill, Liisi Kööts-Ausmees, Jüri Allik, Anu Realo

**Affiliations:** ^1^Department of Psychology, University of Tartu, Tartu, Estonia; ^2^Estonian Academy of Sciences, Tallinn, Estonia; ^3^Department of Psychology, University of Warwick, Coventry, United Kingdom

**Keywords:** anger expression, anger in and anger out, experience sampling, personality, momentary emotions, co-occurring emotions, cross-level interactions

## Abstract

The main aim of the current study was to examine the role of co-occurring emotions and their interactive effects with the Big Five personality traits in anger expression. Everyday anger expression (“anger-in” and “anger-out” behavior) was studied with the experience-sampling method in a group of 110 participants for 14 consecutive days on 7 random occasions per day. Our results showed that the simultaneously co-occurring emotions that buffer against anger expression are sadness, surprise, disgust, disappointment, and irritation for anger-in behavior, and fear, sadness and disappointment for anger-out reactions. While previous studies have shown that differentiating one's current affect into discrete emotion categories buffers against anger expression (Pond et al., 2012), our study further demonstrated the existence of specific interactive effects between the experience of momentary emotions and personality traits that lead to higher levels of either suppression or expression of anger behavior (or both). For example, the interaction between the trait Openness and co-occurring surprise, in predicting anger-in behavior, indicates that less open people hold their anger back more, and more open people use less anger-in behavior. Co-occurring disgust increases anger-out reactions in people low in Conscientiousness, but decreases anger-out reactions in people high in Conscientiousness. People high in Neuroticism are less likely to engage in anger-in behavior when experiencing disgust, surprise, or irritation alongside anger, but show more anger out in the case of co-occurring contempt. The results of the current study help to further clarify the interactions between the basic personality traits and the experience of momentary co-occurring emotions in determining anger behavior.

## The role of co-occurring emotions and personality traits in anger expression

Anger is one of the most common negative emotions and it is experienced about 10% of the time (Trampe et al., 2015). The experience of anger has been described as an emotional reaction to a perceived threat to an individual's emotional well-being (Beck, 1999) and can be summarized as “the experience of something unpleasant and that has obstructed one's reaching one's goals, which event was felt to be unfair but inevitable, and for which someone else is to blame” (Frijda et al., 1995, p. 139). Anger has been conceptualized as a social emotion that emerges in response to the actions of other people (Averill, 1982; Frijda, 1993).

The experience of anger may vary from mild irritation to intense fury, and is accompanied by physical reactions indicating autonomic nervous system arousal (Spielberger, 2010). Previous research has suggested many factors as antecedents to, or moderators of, the anger experience, such as the perception of a threat to, or an injustice against, oneself (Skarlicki and Folger, 1997). The experience of anger may also result from an appraisal of external stimuli as threatening (Huesmann, 1998), or from the perceived violation of a socially acceptable behavior or other social stressor (Berkowitz and Harmon-Jones, 2004). In the context of close relationships, anger can be triggered by a fear of being abandoned, reflecting an underlying continuing desire for connection (Dallos and Vetere, 2009).

The experience of anger, however, must be separated from the expression of anger. Differently from other biologically-based response tendencies (e.g., reflexes), emotions only predispose people to act in a certain manner: they do not force people to do so (Gross and John, 1997). Thus, appraisal of an anger-provoking or frustrating situation triggers the anger experience which, in turn, generates different behavioral responses. In research on anger expression, the distinction between “anger-in” and “anger-out” behavioral reactions is most frequently made. People are classified as “anger in” if they tend to suppress their anger or to direct it toward themselves or “anger out” if they direct their anger outward and express it toward other people or the environment (Funkenstein et al., 1954; Spielberger et al., 1985). Anger-in and anger-out reactions are not conceptualized as opposite poles of one dimension, but rather as two distinct processes that may even have different genetic mechanisms (Guo et al., 2015). The expression of anger is argued to be highly controlled and frequently suppressed or replaced by socially appropriate expressions, and, as a result, only a small proportion of anger experience results in overtly aggressive behavior (Averill, 1983). The purpose of anger-out expression is often to correct injustice, to stand up for oneself, or to change a situation in order not to feel antagonistic emotions. In general, the overt expression of anger may have a negative effect on social interactions, except in some specific situations, such as short term negotiations among strangers (Van Kleef et al., 2004). Anger-in expression, however, often refers to anger suppression for the sake of maintaining good relationships.

What are the factors that lie behind people's reactions if they experience anger? It has been suggested that both anger experience and expression depend on situational (e.g., another person's misdeeds, physical and psychological distress) and dispositional factors (e.g., intrapersonal demands, low agreeableness, high neuroticism); there is, however, little insight into the interplay between the two (Affleck et al., 1999; Mazerolle et al., 2003; Kashdan et al., 2016). It has also been suggested that experiencing one emotion (i.e., anger) can instantly elicit other emotions that interact with the prevailing emotion (Izard, 1972). Emotions are experienced in a mixed way as a response to the contrasting affective qualities of one external event (Russell, 2003). There is also some evidence from anger communication studies that the expression of anger may be influenced by a wide array of situational and contextual factors (Sanford, 2012), but further evidence is needed about the effects of the specific interactions between underlying personality traits and concurrent feelings. Among other things, a better understanding of the influence of the emotional palette co-occurring with anger would be useful for emotion-focused intervention programs. This is why, in the current study, we aim to examine the role of co-occurring emotions and personality traits in anger expression. The use of an experience sampling research design allows us to explore anger behavior in an everyday context, and to include personality traits as individual explanatory characteristics. Below we explain how co-occurring emotions and personality traits might be relevant in anger expression.

### The role of co-occurring emotions in anger expression

There is growing interest in the interplay between emotions that are experienced and the way in which they affect people's behavior. Co-occurring emotions are simultaneously arising emotional states which maintain their discrete characteristics of valence and intensity (Harley et al., 2012). Concurrent feelings about the same object or event are also defined as mixed emotions, usually a pair of emotions with the opposite valence (Berrios et al., 2015). For instance, the expression of emotions has been found to depend on the general affective context, which reflects the emotional experiences of previous periods, averaged across multiple assessments (Sanford, 2012). Previous research has suggested that the experience of anger is frequently blended with the experience of other emotions, such as sadness, fear, disgust, and surprise (Berkowitz and Harmon-Jones, 2004; Trampe et al., 2015). It has also been found that the perception of so-called “hard” emotions (e.g., anger, irritation) expressed during communication dominates co-occurring “soft” emotions (e.g., sadness, disappointment) (Sanford, 2012). In addition, anger has been found to be frequently accompanied with disappointment. Both of these emotions are similar in valence and intensity, but are linked to different appraisals and elicit different behaviors (Lelieveld et al., 2012). Trampe et al. (2015) found that the experience of anger most frequently co-occurs with the feelings of sadness and disgust, while the experience of anger tends to inhibit the experience of joy and other positive emotions. Sanford (2012) reported that the simultaneous experience of soft negative emotions (i.e., sadness) has no effect on the expression of hard emotions (i.e., anger), but the presence of hard emotions affects the expression of soft emotions. Previous studies have also shown an interaction between the expression of anger and the experience of fear, with angry communications themselves inducing fear in a communication partner (Van Kleef et al., 2004). The feeling of fear is related to perceptions of risk (Lerner et al., 2015). A fear of retaliation and an expectation of social disapproval have also been found to inhibit the expression of anger (Bandura, 1973; Berkowitz, 1993; Beck, 1999). It has been shown that induced fear leads to higher levels of anger, and induced sadness leads to lower levels of aggression (Zhan et al., 2015). Additionally, a study on the function of mixed emotions (i.e., two opposing emotions) found that the experience of secondary mixed emotions promotes adaptive coping in stressful situations by lowering the perceived negativity of an adverse event (Davydov et al., 2011), and by supporting solution-oriented actions to handle adversity (Braniecka et al., 2014). For example, concurrent negative emotions in victims of stalking have been found to lead to the use of more effective coping strategies (Ngo and Paternoster, 2013). Thus, experiencing mixed emotions has been suggested to decrease distress felt, to help find meaning in life's stressors (Larsen et al., 2003), and to lead to greater emotional resilience (Tugade and Fredrickson, 2004). In addition, Bosch and D'Mello (2014) found that co-occurring affective states influence success in learning. Berrios et al. (2015) point to the need for further information about different situational activation patterns in mixed affective responses and the functionality of affective co-activation of emotion-related behavior. Although previous studies have implied what the functional effects of co-occurring and mixed emotions are, they cannot be detected in detail unless modeled explicitly. The current study aims to enrich the existing research by examining the role of co-occurring or simultaneously experienced emotions in anger expression.

### The role of personality traits in anger expression

Studying the associations between personality traits and affective states would enable us to detect which personality traits predispose individuals to the expression of anger. Previous research has shown that there are considerable individual differences in the disposition to experience anger (“anger-proneness”) as well as to express it (Jones et al., 2011). State-Trait Anger Theory describes state anger as an immediate subjective experience and trait anger as a personality trait characterized by the tendency to experience frequent state anger (Spielberger, 2010). Accordingly, different constructs, such as trait aggressiveness, trait irritability, and trait anger, Type A personality, dissipation-rumination, impulsivity, and narcissism (Bettencourt et al., 2006) have been examined in relation to the experience and expression of anger. The Big Five personality traits are also often examined with regard to anger experience and expression, with anger experience being typically related to neuroticism and anger expression to agreeableness (Costa et al., 1989). It should be noted, however, that a recent study on anger experience in everyday life did not find any significant associations between the Big Five personality traits and daily anger experiences (Kashdan et al., 2016). A recent study by Pease and Lewis (2015) showed that neuroticism and agreeableness were associated with the trait-level components of the expression of anger, whereas conscientiousness and extraversion were linked to the more focal components of anger expression. In addition, the relationship between neuroticism and the expression of anger was moderated by agreeableness and conscientiousness. It has also been shown that emotion differentiation as a trait moderates aggressive responding by weakening the relationship between anger and aggressive reactions (Pond et al., 2012). Although personality research suggests an interaction between basic personality traits and emotion states (Costa et al., 1992; Pease and Lewis, 2015), almost no attention to date has been paid to interactive influences between personality and momentary emotions on the one hand and anger expression on the other. In the current study, it was predicted that the relationship between co-occurring emotions and anger expression would be moderated by personality traits. As previous studies have stressed the different pathways in anger expression-out vs. expression-in (Pease and Lewis, 2015), we expected emotion-personality moderators to be different for anger-in and anger-out behaviors. However, a more detailed picture of the influence of the specific emotions co-occurring with the anger experience would provide useful information about the exact nature of the links between co-occurring emotions and anger behavior.

### The aim of the study

The main aim of the current study was to examine the role of co-occurring emotions, the Big Five personality traits, and the interaction between these in anger expression. First, based on the earlier research described above (e.g. Pond et al., 2012; Sanford, 2012; Pease and Lewis, 2015; Trampe et al., 2015), we predicted that the experience of fear, sadness, happiness, irritation, surprise, contempt, disgust, and disappointment would uniquely influence anger-in and anger-out behaviors. It was expected that so-called “hard” co-occurring emotions (i.e., irritation) would increase anger-out expression, and “soft” co-occurring emotions increase anger-in expression (i.e., sadness, fear), whereas emotions with the opposite valence (i.e., happiness, surprise) would decrease both anger-in and anger-out behaviors.

Second, we expected that the Big Five personality traits would improve the model fit in explaining anger behavior when also taking into account accompanying momentary emotions. Personality traits have been found to influence anger expression (Jones et al., 2011; Pease and Lewis, 2015), but there is little research to date on whether and which personality traits can explain any additional variance in anger behavior beyond the co-occurrence of anger and other affective states. It was predicted that high neuroticism and low agreeableness predict higher levels of anger expression, and conscientiousness would lead to lower levels of both anger-in and anger-out expressions.

Third, we were interested in examining possible cross-level interactions between the Big Five personality traits and momentary emotional experiences in predicting anger expression in everyday life. Previous studies have highlighted the importance of emotion differentiation in predicting aggressive responses (Pond et al., 2012) and the significance of interactions between personality traits (Pease and Lewis, 2015) and the experience of anger. Interactions may indicate inverse relationships between a predictor (e.g., discrete momentary emotions) and a dependent variable (e.g., anger-in vs. anger-out behavior), when moderated by a third variable (e.g., personality traits).

Taken together, the aim of the present study was to examine how the co-occurrence of momentary anger and other emotions, the Big Five personality traits, and cross-level interactions between the two factors are associated with anger-in and anger-out expressions in daily life.

## Method

This study is a part of a broader research project exploring emotion experience in daily life (see also Kööts et al., 2011, 2012; Mill et al., 2016). The sample consisted of 110 participants (70 females and 40 males) with ages ranging from 19 to 84 years. All participants were ethnic Estonians and received about EUR 33 for taking part in the study. The sample was made up of two subsamples: one of older adults and one of university students. The older participants consisted of 42 females and 13 males and the age of participants in this group ranged from 61 to 84 years, with a mean age of 68.2 (*SD* = 5.5). The majority (73%) of the respondents was retired; about one-third (36%) of older respondents had higher education. The student group (*n* = 55; 28 females and 27 males) was made up of undergraduates from the University of Tartu; those majoring in psychology were not eligible to participate. The mean age of students was 21.3 (*SD* = 1.0), ranging from 19 to 23 years.

### Procedure

The study consisted of 14 days of experiment by the experience sampling method (ESM) using iESP software. Participants were signaled 7 times per day (the time point was randomly chosen by the iESP software) during the average waking time from 8 a.m. to 8 p.m. to report their current emotions (i.e., up to 98 possible assessments per participant). There were 10,667 measurement trials across all participants, with an average of 97 measurement trials per participant. The response rate was 82.8%, which is considered to be in the normal range for an experience-sampling study (Zelenski and Larsen, 2000).

### Measures

#### State-level measures

Participants were asked to indicate on a 4-point Likert-type scale (1–*not at all* to 4–*to a large extent*), as used in other ESM studies (Mroczek et al., 2003; Gerstorf et al., 2009; Thompson et al., 2011), the extent to which each of the measured emotions (anger, happy, contempt, disgust, fear, sadness, disappointment, irritation and surprise) described their current emotional state as quickly and accurately as possible.

The expression of anger was measured by the following three statements. When I was angry, (1) I kept the anger inside me; (2) I expressed my anger; (3) I tried to maintain self-control (The three options describe anger in, anger out, and anger control, respectively). All three statements were answered on a four-point scale from 1–*not at all* to 4–*to a large extent*. In the current study, the anger-in and anger-out behaviors are the main factors of interest.

#### Trait-level measures

At the beginning of the experiment, participants filled in the Estonian version (Kallasmaa et al., 2000) of the Revised NEO Personality Inventory (NEO-PI-R; Costa et al., 1992). The NEO PI-R consists of 240 items that measure five broad factors—Neuroticism, Extraversion, Openness to Experience, Agreeableness, and Conscientiousness—and their 30 facets. Each facet is measured by 8 items, and items are answered on a 5-point Likert scale, ranging from 0 *(strongly disagree)* to 4 *(strongly agree)*.

### Analyses

As anger behavior episodes are nested within individuals, the data were analyzed by way of a multilevel regression analysis (Nezlek, 2007). The experience of anger and its co-occurring emotions, as well as the expression of anger, are measured at level 1 (state level), whereas personality traits are measured at level 2 (trait level). Methodologically, the aim was to carry out a multilevel regression analysis of the two distinct types of anger expression (anger in vs. anger out), by including the experience of anger and other momentary emotions as Level 1 explanatory variables, and personality traits as Level 2 measures. The multilevel model makes it possible to establish the increase in the explanation of individual differences in state anger implied by Level 2 factors. Also, it is possible to determine the specific contribution of different explanatory factors and interactions. More specifically, the aim of the multilevel regression analysis was to find the best predictors for the two types of anger expression, but also to determine what set of variables comprises the best model fit, providing the best explanation for momentary anger-in and anger-out behaviors (the parsimony of the different independent variables). The model included anger-in and anger-out behaviors as outcome variables; predictor variables consisted of momentary ratings of the experience of co-occurring emotions and their interactions with momentary anger (Level 1 predictors), and personality traits, and age were entered as Level 2 predictors. As is common in the predictive approach, variables were retained in the model on the basis of statistical significance and model efficiency (Heck et al., 2010). To assess improvement in model fit by comparing three successive models, maximum likelihood estimation was used (Heck et al., 2010). A series of multilevel models was conducted in the Mixed module of IBM SPSS 20.0: first the null-model was created, followed by the Level 1 predictors model (momentary emotions), and the Level 2 predictors model (personality and age) including cross-level interactions as predictors. In the equations of multilevel models predicting anger behavior, the (*i*) refers to momentary recordings of anger behavior and (*j*) refers to each individual, as the observations were nested within participants. The anger behavior *i* of participant *j* can be represented as follows, with β_0j_ as the intercept and ε_*ij*_ as variation in estimating the momentary anger behavior of participants:

$$\begin{array}{l} Y_{\text{ij}}=\beta_{0\text{j}}+\varepsilon_{\text{ij}}\\ \beta_{0\text{j}}=\gamma_{00}+u_{0\text{j}}. \end{array}$$

The null model, providing the estimated mean anger behavior for all participants and partitioning variance between Level 1 (ε_*ij*_*)* and Level 2 (*u*_0*j*_), is represented as:

$$\begin{array}{l} Y_{\text{ij}}=\gamma_{00}+u_{0\text{j}}+\varepsilon_{\text{ij}} \end{array}$$

Next, a Level 1 model was built with momentary emotions (i.e., anger plus the other six basic emotions) as within-person predictors of anger behaviors. The within-person random intercept model was defined as follows, suggesting that, at the within-person level, anger behavior is related to experienced momentary emotions:

Y_ij_ = β_0j_ + β_1_(anger)_ij_ + β_2_(fear)_ij_ + β_3_(sadness)_ij_ + β_4_(surprise)_ij_ + β_5_(disgust)_ij_ + β_6_(disappointment)_ij_ + β_7_(contempt)_ij +_ β_8_(irritation)_ij_ + β_9_(happiness)_ij_ + β_10_(anger^*^fear)_ij_ + β_11_(anger^*^sadness)_ij_ + β_12_(anger^*^surprise)_ij_ + β_13_(anger^*^disgust)_ij_ + β_14_(anger^*^disappointment)_ij_ + β_15_(anger^*^contempt)_ij_ + β_16_(anger^*^irritation)_ij +_ β_17_(anger^*^happiness)_ij_ + εij.

As the research model of the current study proposes that the relationship between momentary emotions and anger behavior may vary across individuals, a person-level random intercept model was built, based on the assumption that dispositional factors (i.e., personality traits and age) impact the remaining variability in anger behavior between people. At the between-person level, the facets of the Big Five personality traits and age were added to the model to explain the remaining variability in anger behaviors between individuals:

β_0j_ = γ_00_ + γ_01_(fear) _ij_ + γ_02_(sadness)_*ij*_ + γ_03_(happiness)_*ij*_ + γ_04_(surprise)_ij_ + γ_05_(contempt)_ij_ + γ_06_(disgust)_ij_ + γ_07_(disappointment)_*ij*_ + γ_08_(irritation)_*ij*_ + γ_09_(anger)_ij_ + γ_10_(anger^*^fear)_*ij*_ + γ_11_(anger^*^sadness)_*ij*_ + γ_12_(anger^*^surprise)_*ij*_ + γ_13_(anger^*^disgust)_*ij*_ + γ_14_(anger^*^disappointment)_*ij*_ + γ_15_(anger^*^contempt)_*ij*_ + γ_16_(anger^*^irritation)_ij +_ γ_17_(anger^*^happiness)_*ij*_ + γ_18_(neuroticism)_j_ + γ_19_(extraversion)_*j*_ + γ_20_(openness to experience)_*j*_ + γ_21_(agreeableness)_*j*_ + γ_22_(conscientiousness)_*j*_ + γ_23_(age)_*j*_ + u_0j_.

For all analyses, the control variable of age was used, as it has been shown that emotion behaviors can be associated with aging effects (John and Gross, 2004; Mill et al., 2009). In terms of centering, grand mean centering (GMC) was used for all person-level variables, and, for continuous state-level variables, group mean centering (CWC) was used (Enders and Tofighi, 2007; Nezlek, 2007).

## Results

### The frequency of the experience of anger

The experience of anger (altogether recorded 660 times) was as follows: 475 incidences of slight anger (2–*to some extent*), 138 incidences of moderate anger (3–*to a moderate extent*), and 47 incidences of strong anger (4–*to a large extent*), *M* = 2.27 (*SD* = 0.32). On average, anger was experienced 5.98 times per person during the 2 weeks (*SD* = 5.62, ranging from 1 to 30 anger occasions).

First, the frequencies of experienced anger and other emotions were computed as the percentage of the time that people reported feeling an emotion. On average, people reported experiencing anger on 6.19%, fear on 8.13%, sadness on 21.50%, surprise on 22.76%, disgust on 8.21%, happiness on 67.95%, disappointment on 19.90%, contempt on 6.93%, and irritation on 25.95% of all measurement occasions. In general, the amount of time experiencing specific emotions in everyday life was similar to that reported in a previous study by Trampe et al. (2015).

We were also interested of co-occurrence of different emotions, referring to the frequency of situations in which participants rated the experience of respective emotions greater than 1 (*not at all*). The frequencies of co-occurring emotions when anger was experienced (660 episodes) were the following: fear 24.09%, sadness 58.79%, surprise 37.27%, happiness 46.36%, disgust 35.44%, disappointment 67.42%, contempt 33.03% and irritation 77.27%. The extent of co-occurrence of mixed and same valence emotions was at similar level as reported by previous studies for the mixed experience of sadness and happiness (Larsen et al., 2001; Moeller et al., 2018). When experiencing anger, people used anger-in behavior on 86% (568 cases) and anger-out behavior on 60% (393 cases) of measurement occasions; thus, anger is usually both held back and expressed (the correlation between anger-in and anger-out expressions was as high as *r* = 0.78, *p* < 0.001). The correlations between the experience of anger and anger-in/anger-out expressions were *r* = 0.84 and 0.85, respectively (*p* < 0.001). The descriptive statistics of predictor and dependent variables are shown in Appendix 1 in Supplementary Material.

### Anger behavior explained by experienced momentary emotions and personality traits

Next, we examined the effects of the co-occurrence of anger and other emotions and the Big Five personality traits on anger expression (in vs. out). In addition, we explored the mechanisms underlying the interaction effect of personality traits on the relationship between co-occurring momentary emotions and anger behavior.

To this end, a series of multilevel regression analyses were conducted.

In the first step of the multilevel regression analysis, we examined whether anger behaviors varied across people. The residual parameters of the no predictors model, describing the variance to be explained within groups, suggested that there was a significant variance to be explained at the within-person level both for anger-in and anger-out expression (*Z* = 65.97, *p* < 0.001 and *Z* = 65.97, *p* < 0.001, respectively). Also, the intercept parameters indicated that the intercepts varied significantly across people (*Z* = 6.14, *p* < 0.001, and *Z* = 6.09, *p* < 0.001, respectively for anger-in and anger-out expression). Thus, people differed from one another as well as varied within themselves in anger behavior, and we expected to understand these differences by exploring the effects of momentary and trait-level predictors.

The results of mixed models analysis, in terms of significance of predictors, are presented in Table 1.

**Table 1:** The null model and Level 1 fixed effects model of anger-in and anger-out behaviors.

**Model**	**Predictors**	**Anger-in**					**Anger-out**				
		**Estimates of fixed effects**					**Estimates of fixed effects**				
		**Estimate**	**Std. Error**	***df***	***t***	**Sig**.	**Estimate**	**Std. Error**	***df***	***T***	**Sig**.
Null model	Intercept	0.22	0.02	108	10.52	**0.000**	0.15	0.01	110	10.32	**0.000**
Level 1 predictors	Intercept	0.25	0.02	109	10.44	**0.000**	0.15	0.02	109	10.28	**0.000**
	Fear	0.01	0.01	8,687	1.12	0.262	−0.02	0.01	8,687	−2.01	**0.045**
	Sadness	0.01	0.01	8,687	0.61	0.540	−0.01	0.01	8,687	−0.82	0.410
	Happiness	−0.01	0.01	8,687	−2.53	**0.011**	−0.01	0.00	8,687	−1.89	0.059
	Surprise	0.00	0.01	8,687	−0.57	0.566	0.01	0.01	8,687	1.46	0.143
	Contempt	0.03	0.01	8,687	1.78	0.075	0.01	0.01	8,688	0.52	0.600
	Disgust	0.02	0.01	8,689	1.51	0.130	0.02	0.01	8,689	1.70	0.089
	Disappointment	0.04	0.01	8,687	4.26	**0.000**	0.01	0.01	8,687	2.20	**0.028**
	Irritation	0.04	0.01	8,688	4.05	**0.000**	0.05	0.01	8,688	7.39	**0.000**
	Anger	2.05	0.02	8,710	120.53	**0.000**	1.27	0.01	8,717	105.28	**0.000**
	Fear^*^Anger	−0.01	0.02	8,701	−0.31	0.754	−0.09	0.01	8,705	−6.22	**0.000**
	Sadness^*^Anger	−0.04	0.02	8,700	−2.34	**0.019**	−0.08	0.01	8,705	−7.34	**0.000**
	Happiness^*^Anger	−0.02	0.02	8,700	−1.17	0.243	−0.02	0.01	8,704	−1.48	0.139
	Surprise^*^Anger	−0.08	0.01	8,695	−5.34	**0.000**	0.02	0.01	8,698	1.72	0.086
	Contempt^*^Anger	0.01	0.02	8,696	0.67	0.502	0.00	0.01	8,699	−0.14	0.885
	Disgust^*^Anger	−0.19	0.02	8,702	−10.26	**0.000**	−0.07	0.01	8,707	−5.59	**0.000**
	Disappointment^*^Anger	0.07	0.02	8,698	4.77	**0.000**	−0.03	0.01	8,702	−2.29	**0.022**
	Irritation^*^Anger	−0.35	0.01	8,708	−23.47	**0.000**	0.00	0.01	8,715	−0.21	0.831

*Bold values outline the significant results*.

The intercepts adjusted for Level 1 predictors were *Y* = 0.25 (*p* < 0.001) for anger-in and *Y* = 0.15 (*p* < 0.001) for anger-out expression. The experience of anger [β_1_ = 2.05, *t*_(8, 710)_ = 120.53, *p* < 0.001], disappointment [β_6_ = 0.04, *t*_(8, 687)_ = 4.26, *p* < 0.001], irritation [β_8_ = 0.04, *t*_(8, 688)_ = 4.05, *p* < 0.001] and momentary happiness [β_6_ = −0.01, *t*_(8, 687)_ = −2.53, *p* < 0.05], significantly predicted anger-in expression. This suggests that anger-in behavior is greater given the moment-to-moment increases in momentary disappointment and irritation over and above that is predicted by anger. Whereas, in case of co-occurring happiness there is less cognitive effort in holding anger inside oneself. However, were also interested of within-person interactions of momentary emotions (Level-1 predictors) in order to assess the effects of emotions that co-occur with anger on anger-in and anger-out expression. There were following significant interactions between anger and co-occurring emotions of sadness [β_11_ = −0.04, *t*_(8, 700)_ = −2.34, *p* < 0.05], surprise (β_12_ = −0.08, *t*_(8, 685)_ = −5.34, *p* < 0.001], disgust [β_13_ = −0.19, *t*_(8, 703)_ = −10.28, *p* < 0.001], disappointment [β_14_ = 0.07, *t*_(8, 699)_ = 4.76, *p* < 0.001], and irritation [β_16_ = −0.35, *t*_(8, 708)_ = −23.47, *p* < 0.001], in predicting anger-in behavior. Thus, the stronger the experience of anger, the more one tries to hold it back. In addition, the co-occurrence of several other emotions with anger seems to influence anger-in behavior—people are more likely to use anger-in behavior when feeling not only angry but also disappointed, whereas the co-occurrence of anger and sadness, surprise, irritation, or disgust leads to lesser engagement in anger-in behavior.

For anger-out expression, the significant predictors were the momentary experience of anger [β_1_ = 1.27, *t*_(8717)_ = 105.28, *p* < 0.001], irritation [β_8_ = 0.05, *t*_(8688)_ = 7.39, *p* < 0.001], fear [β_2_ = −0.02, *t*_(8687)_ = −2.01, *p* < 0.05], and disappointment [β_6_ = 0.01, *t*_(8687)_ = 2.20, *p* < 0.05]. There were also significant interactions between anger and following co-occurring emotions in predicting anger-out behavior: fear [β_10_ = −0.09, *t*_(8705)_ = −6.22, *p* < 0.001], sadness [β_11_ = −0.08, *t*_(8705)_ = −7.34, *p* < 0.001], disgust [β_13_ = −0.07, *t*_(8707)_ = −5.59, *p* < 0.001], and disappointment [β_14_ = −0.03, *t*_(8702)_ = −2.29, *p* < 0.001]. Thus, simultaneously experienced emotions have a significant influence on anger-out behavior–anger is less overtly expressed when the experience of anger is accompanied by fear, sadness, disgust, and disappointment. Interestingly, the direction of the effect of disappointment on anger-out reaction depends on levels of simultaneously experienced anger. Taken together, co-occurring emotions may influence anger-in and anger-out behaviors in different ways. Sadness and disgust, for example, make one hold anger expression back less, but also decrease the anger-out reaction. Disappointment, increases efforts to hold back anger, and also decreases anger-out reactions. Irritation decreases anger-in behavior, whereas sadness decreases only anger-out reactions.

The addition of within-person predictors did not reduce the within-group variability in anger-in and anger-out behaviors significantly. The intraclass correlation (ICC) as the ratio of between-groups variance to the total variance was also calculated (Heck et al., 2010). The ICC was ρ = 0.28 for anger-in, and ρ = 0.23 for anger-out, expression. There was considerable change in likelihood function between the null model and the level 1 model for anger-out behavior, suggesting that Level 1 predictors improved the model fit:−2^*^LL (-2^*^Log Likelihood) decreased from 20901 to 9600 for anger-in, and from 14488 to 3506 for anger-out, models. Yet, there was still significant variability to be explained both within and between people (at *p* < 0.001). This suggested that it would be meaningful to add person-level predictors that could explain this remaining residual variability in intercepts.

The addition of Level 2 predictors did not improve the models, the−2^*^LL was increased from 9600 to 9644 for anger-in and from 3506 to 3562 for anger-out behavior. The results of the mixed models analysis with fixed predictors are presented in Table 2.

**Table 2:** The estimates of the Level 2 fixed effects model of anger-in and anger-out behaviors.

**Model**	**Predictors**	**Anger-in**					**Anger-out**				
		**Estimates of fixed effects**					**Estimates of fixed effects**				
		**Estimate**	**Std. Error**	***df***	***T***	**Sig**.	**Estimate**	**Std. Error**	***df***	***t***	**Sig**.
Level 2 predictors model	Intercept	0.25	0.02	103	11.57	**0.000**	0.15	0.01	103	11.05	**0.000**
	Fear	0.01	0.01	8,687	1.13	0.260	−0.02	0.01	8,687	−2.00	**0.045**
	Sadness	0.01	0.01	8,687	0.61	0.543	−0.01	0.01	8,687	−0.83	0.408
	Happiness	−0.01	0.01	8,687	−2.53	**0.011**	−0.01	0.00	8,687	−1.89	0.059
	Surprise	0.00	0.01	8,687	−0.57	0.567	0.01	0.01	8,687	1.47	0.142
	Contempt	0.03	0.01	8,687	1.78	0.076	0.01	0.01	8,688	0.52	0.602
	Disgust	0.02	0.01	8,689	1.52	0.128	0.02	0.01	8,689	1.71	0.087
	Disappointment	0.04	0.01	8,687	4.26	**0.000**	0.01	0.01	8,687	2.20	**0.028**
	Irritation	0.04	0.01	8,688	4.07	**0.000**	0.05	0.01	8,688	7.41	**0.000**
	Anger	2.05	0.02	8,707	120.56	**0.000**	1.27	0.01	8,712	105.32	**0.000**
	Fear^*^Anger	−0.01	0.02	8,703	−0.33	0.739	−0.09	0.01	8,707	−6.25	**0.000**
	Sadness^*^Anger	−0.04	0.02	8,702	−2.32	**0.021**	−0.08	0.01	8,705	−7.34	**0.000**
	Happiness^*^Anger	−0.02	0.02	8,701	−1.15	0.250	−0.02	0.01	8,704	−1.47	0.142
	Surprise^*^Anger	−0.08	0.01	8,696	−5.34	**0.000**	0.02	0.01	8,699	1.72	0.086
	Contempt^*^Anger	0.01	0.02	8,697	0.70	0.483	0.00	0.01	8,699	−0.11	0.909
	Disgust^*^Anger	−0.19	0.02	8,703	−10.28	**0.000**	−0.07	0.01	8,707	−5.61	**0.000**
	Disappointment^*^Anger	0.07	0.02	8,699	4.76	**0.000**	−0.03	0.01	8,701	−2.31	**0.021**
	Irritation^*^Anger	−0.35	0.01	8,707	−23.53	**0.000**	0.00	0.01	8,712	−0.28	0.783
	Neuroticism	0.00	0.00	102	1.67	0.098	0.00	0.00	102	2.03	**0.045**
	Extraversion	0.00	0.00	103	2.35	**0.021**	0.00	0.00	103	1.94	0.055
	Openness to Experience	0.00	0.00	102	−0.02	0.983	0.00	0.00	102	0.45	0.651
	Agreeableness	0.00	0.00	102	−2.31	**0.023**	0.00	0.00	102	−2.99	**0.003**
	Conscientiousness	0.00	0.00	103	−2.22	**0.029**	0.00	0.00	103	−0.96	0.337
	Age	0.00	0.00	102	0.49	0.626	0.00	0.00	102	1.12	0.267

*Bold values outline the significant results*.

Regarding the predictors, anger-in behavior was influenced by Extraversion [γ_19_ = 0.003, *t*_(103)_ = 2.35, *p* < 0.05], Agreeableness [γ_21_ = −0.003, *t*_(102)_ = −2.31, *p* < 0.05] and Conscientiousness [γ_22_ = −0.003, *t*_(103)_ = −2.22, *p* < 0.05]. Significant predictors for anger-out were Neuroticism [γ_18_ = 0.001, *t*_(102)_ = 2.03, *p* < 0.05] and Agreeableness [γ_21_ = −0.003, *p* < 0.01]. The main effects for both anger-in and anger-out Level 2 models were statistically significant at *p* < 0.001, suggesting that anger-in and anger-out behaviors vary significantly as a function of co-occurring emotions and personality traits.

### Moderating effects of personality traits on the association between co-occurring emotions and anger behavior

One of the main predictions of our study was that personality traits act as moderating factors on the relationship between co-occurring emotions and personality traits. It is possible that co-occurring emotions influence anger expression in a certain way for people with specific levels of personality traits. In order to investigate this, two-way interactions with a simple slope analysis between momentary emotions and personality traits were added to the Level 2 model. Interactions were probed across values of moderator variables according to techniques described by Dawson (2014).

**Figure 1:**
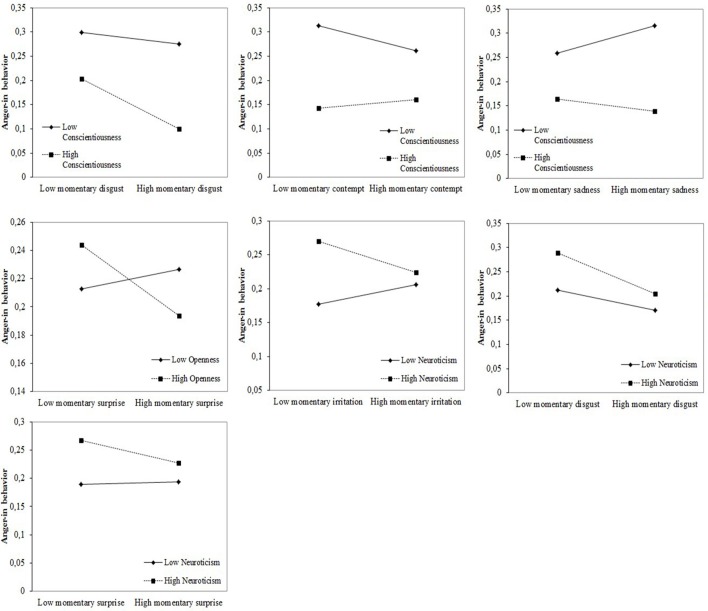
Statistically significant interactions between momentary emotions and personality traits in predicting anger-in behavior.

**Figure 2:**
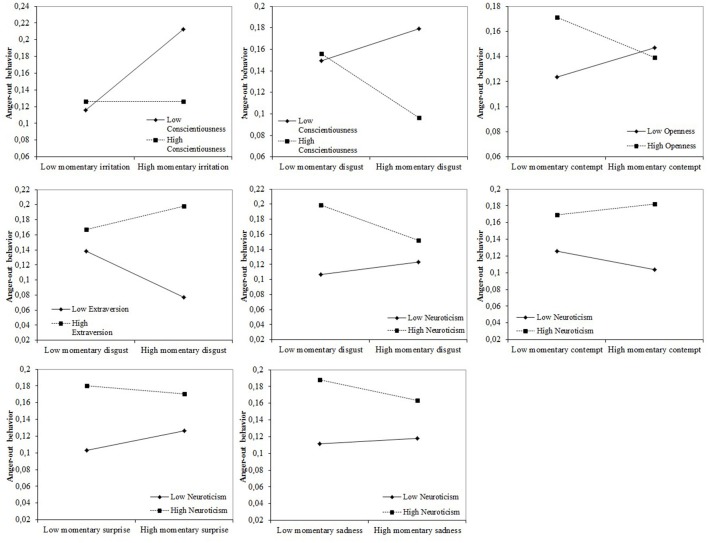
Statistically significant interactions between momentary emotions and personality traits in predicting anger-out behavior.

As expected, the analyses revealed statistically significant Momentary Co-occurring Emotion x Personality interactions for both anger-in and anger-out behaviors, as presented in Figures 1, 2.

A variety of interaction effects between momentary emotions and personality traits emerged for both anger-in and anger-out behaviors (Reinard, 2006). For anger-in behavior, there was an interaction between Conscientiousness and disgust, with people high in Conscientiousness using less anger-in behavior when feeling both angry and disgusted [ŷ = −0.002, *t*_(8, 704)_ = −2.54, *p* = 0.011]. There was also an interaction between Conscientiousness and contempt—people low in Conscientiousness tended not to hold back anger when feeling contempt, whereas people high in Conscientiousness rather held their anger in, in the case of co-occurring contempt [ŷ = 0.002, *t*_(8, 704)_ = 2.22, *p* = 0.027]. The co-occurrence of sadness, however, made people low in Conscientiousness not hold anger in, whereas, for people high in Conscientiousness, there was the opposite effect [ŷ = −0.001, *t*_(8, 704)_ = −2.87, *p* = 0.004; see Figure 1]. There was also an interaction effect between Openness and surprise, with people high in Openness holding less anger in when feeling momentary surprise, whereas for people low in Openness, the experience of momentary surprise made them more engaged in anger-in behavior [ŷ = −0.001, *t*_(8, 704)_ = −2.40, *p* = 0.016]. There was an interaction effect between Neuroticism and irritation, with people high in Neuroticism holding less anger in when not being irritated at the time, whereas people low in Neuroticism showed more anger-in behavior when also experiencing irritation [ŷ = −0.001, *t*_(8, 704)_ = −3.15, *p* = 0.002]. People high in Neuroticism hold anger less in when feeling both disgust [ŷ = −0.001, *t*_(8, 704)_ = −2.05, *p* = 0.041] and surprise [ŷ = −0.002, *t*_(8, 704)_ = −2.24, *p* = 0.025].

For anger out, there were also significant interactions between co-occurring momentary emotions and personality traits in determining anger reactions. There were interactions between conscientiousness and the momentary emotions of irritation [ŷ = −0.002, *t*_(8, 704)_ = −4.53, *p* < 0.001] and disgust [ŷ = −0.002, *t*_(8, 704)_ = −4.35, *p* < 0.001], with high Conscientiousness attenuating the link between irritation and anger-out reactions and disgust increasing anger-out reactions in people low in Conscientiousness, but decreasing anger-out reactions in people low in Conscientiousness. There was a similar interaction effect between Extraversion and disgust [ŷ = 0.002, *t*_(8, 704)_ = 4.01, *p* < 0.001], the co-occurring feeling of disgust making introverted people not engage in anger-out behavior, whereas the effect was the opposite for more extraverted people. There was also an interaction between Openness and contempt [ŷ = −0.001, *t*_(8, 704)_ = −2.63, *p* = 0.008], where the feeling of contempt reduced anger-out expression for people high in Openness. The influence of co-occurring momentary disgust is also moderated by Neuroticism [ŷ = −0.001, *t*_(8, 704)_ = −4.52, *p* < 0.001], with people high in Neuroticism having lower levels of anger-out behavior when accompanied by disgust. More neurotic people have higher levels of anger-out behavior in the case of contempt [ŷ = 0.001, *t*_(8, 704)_ = 2.40, *p* = 0.017], and less anger out when feeling surprise [ŷ = −0.001, *t*_(8, 704)_ = −2.47, *p* = 0.014] or sadness [ŷ = −0.002, *t*_(8, 704)_ = −2.06, *p* = 0.039].

## Discussion

The main objective of the current study was to capture and explore the functional role of co-occurring emotions, personality traits, and the interaction of these in explaining the anger behavior (anger in and anger out) in people's everyday lives. By using an experience sampling approach, we conducted a comprehensive examination into how discrete momentary emotions are related to anger behavior. The results suggest that some co-occurring emotions are important predictors of both anger-in and anger-out behaviors, such that disgust, for instance, reduces both anger-in and anger-out reactions. Thus, it can be that when one is feeling disgusted together with anger, there is less cognitive effort directed to not displaying one's feelings, and the overt display of anger is also weaker. The pattern is similar for sadness. Disappointment, however, has different effect, meaning that when people are not only angry but also disappointed, they are more likely to direct their anger inwards and less outwards. Also, the co-occurring emotions of sadness or irritation result in a stronger anger-in behavior, making it harder to deal with experienced anger. Whereas co-occurring fear reduces just the overt expression of anger.

### Co-occurrence of emotions and anger behavior

Anger is a multifaceted construct that can be experienced and expressed in a various ways, and anger behavior can be expected to be modified and influenced by different trait- and state-level interactions (Pease and Lewis, 2015). Previous studies have suggested that mixed emotions are felt about 33% of time, and there is interplay between emotions that are experienced simultaneously, where some emotions stimulate same-valence emotions and inhibit opposite-valence emotions (Trampe et al., 2015). Regarding anger expression, a study by Sanford (2012) suggested that only co-occurring “hard” negative emotions (averaged across different emotions, including irritation) have an effect on anger expression. However, our study aimed to explore the effect of specific co-occurring emotions on anger-in and anger-out reactions separately. Our analyses suggest that the pattern of influence of simultaneously felt negative emotions is more detailed and may lead to both anger expression and inhibition. Anger is less overtly expressed (anger out) when accompanied by feelings of disappointment, disgust, fear and sadness. Anger out is higher for people high in Neuroticism and low in Agreeableness. More neurotic people report expressing more, and more agreeable people less, anger out. People higher in Extraversion report trying to hold back their anger (anger-in), whereas people high in Agreeableness and Conscientiousness report less effort in doing so. Thus, it appears that personality traits have a direct influence on anger in everyday life over and above momentary emotions, with distinct profiles for the effects on anger in vs. anger out. Our findings broadly support previous studies suggesting that there are dispositional factors that influence the etiology of anger experience and expression, personality traits of Neuroticism, Agreeableness, Extraversion and Conscientiousness can be seen as temporally stable psychobiological basis of behavior. Although a connection between personality traits and anger has been suggested in previous studies (Hofmans et al., 2008; Jones et al., 2011), a recent daily diary study by Kashdan et al. (2016) did not find a link between personality traits and the regulation of everyday anger.

The conclusions of our study are partly in accordance with the findings of a previous study by Sanford (2012): our results similarly indicate that the co-occurring hard emotion of irritation influences anger behavior by reducing anger-in reaction. However, in terms of the soft negative emotions, Sanford (2012) concluded that the presence of these has little influence on the expression of hard emotions. The results of our study clearly show that sadness and disappointment, which were included as soft emotions in Sanford's (2012) study, have a significant influence on anger expression, with disappointment increasing the effort required in holding anger back and decreasing the strength of the anger expression. It has been suggested that communicating disappointment instead of expressing anger has more positive consequences in social communication (Wubben et al., 2011). Disappointment is conceptualized to be felt as a response to unfulfilled positive expectations (an appraisal of self-blame for creating too high expectations), and is associated with a tendency to do nothing (Van Dijk and Van Harreveld, 2008; Reisenzein, 2009). Our results show that even the feeling of disappointment has an effect on anger communication. Thus, our study also suggests that co-occurring emotions should be considered in a detailed way, rather than as simply falling into the two categories of “soft” and “hard” negative emotions. Regarding the influence of specific emotions, surprise, disgust, sadness, disappointment, and fear have a significant effect on anger reactions not explained by personality traits. The direct role of these emotions in buffering anger behavior is in line with previous studies about the experience of mixed emotions, suggesting that there is a constant interplay between simultaneously felt emotions (Trampe et al., 2015). Taken together, our results demonstrate that the understanding of the effect of mixed emotions can be broadened to also include an influence on anger behavior.

### Interaction between co-occurred emotions and personality traits in predicting anger behavior

Although anger is an emotion that is experienced and expressed quite frequently by most people during everyday life, the general proneness to angry feelings has been found to differ widely across individuals (Kuppens et al., 2007). In the current study we observed evidence for moderation between co-occurring emotions and personality traits. Recent study by Moeller et al. (2018) has shown that specific positive and negative emotions tend to occur together during daily life and people are often feeling different and opposite valence emotions at the same time. It is important to understand the effects of co-occurrence of emotions. In our study, the emotions simultaneously felt together with anger had a moderating effect on the pathway between personality and anger expression. It was found that anger is less held back (anger in) when accompanied by happiness and disgust, and held back more when accompanied by disappointment. At the dispositional level, people with higher Extraversion and lower Agreeableness and Conscientiousness report more anger-in expression. Additionally, interactions between personality traits and momentary emotions indicate that the influence of some emotions (i.e., disgust, contempt, sadness, surprise, and irritation) is moderated by personality traits.

The effect of the co-occurrence of certain emotions with anger on anger expression, however, is moderated by specific personality traits, with some emotions increasing anger-in or anger-out reactions only in the case of specific trait levels (i.e., irritation increases anger-out reactions only in people low in conscientiousness). For some emotions, the effect is stronger in the case of specific trait levels (i.e., people tend to hold their anger in less when it is accompanied by disgust, but the effect is stronger for people high in conscientiousness, and there is a similar interaction between disgust and neuroticism). In some cases however, there is an antagonistic moderating effect, suggesting that the effect of emotion on anger behavior is high vs. low depending on personality trait levels (i.e., people high in Extraversion show more anger out compared to people low in Extraversion, but the effect is much stronger in the case of co-occurring disgust). In addition, the effect of co-occurring emotion on anger behavior can be reduced by an interaction with personality (i.e., people high in Openness express more anger out compared to people low in Openness, but that difference is reduced in the case of co-occurring contempt). Thus, the results are the first to demonstrate interactions between the co-occurring emotions and personality traits that influence anger behavior, and these interactions are different for anger-in and anger-out behaviors. Taken together, previous studies have suggested that emotion differentiation buffers against anger expression (Pond et al., 2012). Our study provides more detailed evidence of how co-occurring feelings together with personality traits differentially influence anger behavior in everyday life. This means that stable traits and variable states together create the emotional reaction during anger-inducing situations. Possible mechanisms underlying the obtained results are related to emotion processes and situations when blends of same or different valence emotions are elicited (Gonzalez et al., 2017). Appraisal theories argue that emotion episode is a dynamic process during which the organism evaluates the events and consequences in a series of appraisal checks that results in a unique, context- and individual-specific feeling state (Scherer, 2013). The co-occurrence of different emotions can be explained by differing or even contradictory evaluations given during the appraisal checks. In the context of our results, the co-occurring emotions reflect the context can be expected to be related to relevance and implication assessments. Whereas, personality traits can be expected to influence the coping potential determination and normative significance evaluations that take place during the appraisal process of experiencing emotions. These appraisal results drive the response patterning of intra- or inter-directed anger expression.

## Conclusions, implications, limitations

While much of the research to date addressing the links between personality traits and anger has focused on between-person analyses, almost no attention has focused on whether personality traits show interactive influences with momentary emotions in terms of their effects on anger behavior. The model of the Big Five personality traits (Watson, 2000) as well as models of anger (Wilkowski and Robinson, 2008; DeWall et al., 2011) explicitly contain interactive elements between the two phenomena. Two main findings emerged in current study, which can serve to fill this gap in the literature. First, the co-occurrence of anger and disgust, fear, sadness and disappointment has a direct influence on anger behavior. The second main finding was that there are complex interactions between the momentary emotions of anger and disgust, contempt, surprise, sadness, and irritation, and the Big Five personality traits on anger expression. This demonstrates the complexity of the predictors of anger behavior in people's daily lives. In addition, our study also supported previous studies (Guo et al., 2015), suggesting that although anger in and anger out are clearly related emotion processes, there are also significant differences between the two, by demonstrating that anger in and anger out are influenced by different co-occurring emotions and emotion-personality interactions. Taken together, the current study is the first to date to show how multifaceted anger behavior in the real-world setting is. It is not the feeling of anger that makes one behave angrily, but rather it is the complex interplay of momentary emotions and personality traits.

The results have important implications for psychological interventions aiming to influence anger behavior. Anger behavior is a common problem not only in intimate relationships, but also in workplace communications, and there are different treatment programs aimed at dealing with anger (Wilkowski and Robinson, 2010). It is has been argued that there is a need to expand what is offered in anger management courses, which typically consist of psychoeducation, understanding anger appraisals and triggers, the experiences of anger, and its consequences (Illman and Brown, 2016). Our study suggests that anger management programs could also include an analysis of the emotions experienced simultaneously with anger and take into account people's personality traits at the facet level in order to understand their emotional reactions.

Lastly, it is important to consider the limitations of the current study. The use of self-reports in anger research may be influenced by social desirability and they depend on how people interpret the anger experience, their level of insight into their behavior, and the overall interpretation of the term “anger.” In addition, the sample consisted of two age-groups, future studies could also include middle-aged subjects. Also, the use of single-item measures, not capturing the full spectrum of a construct, can be considered as a limitation of current study, and further research could expand the results using multi-item measures of anger expression.

Nevertheless, despite these limitations, we believe that our study helps reveal the interplay between the momentary emotions and personality dispositions behind anger behavior, and shows the advantages of including a range of co-occurring emotional states and dispositional traits in any effort at understanding daily anger.

## Ethics statement

The Research Ethics Committee of the University of Tartu approved the study, and all participants provided written informed consent. All the research procedures were conducted in accordance with the Declaration of Helsinki.

## Author contributions

Conception and design of the work, the acquisition, analysis, and interpretation of data for the work: AM, LK-A, JA, and AR. Drafting the work or revising it critically for important intellectual content: AM, LK-A, JA, and AR. Final approval of the version to be published: AM, LK-A, JA, and AR.

### Conflict of interest statement

The authors declare that the research was conducted in the absence of any commercial or financial relationships that could be construed as a potential conflict of interest.

## References

[B1] Affleck G. Zautra A. Tennen H. Armeli S. (1999). Multilevel daily process designs for consulting and clinical psychology: a preface for the perplexed. J. Consult. Clin. Psych. 67, 746–754. 10535241 10.1037//0022-006x.67.5.746

[B2] Averill J. (1983). Studies on anger and aggression: implications for theories of emotion. Am. Psychol. 38, 1145–1160. 6650969 10.1037//0003-066x.38.11.1145

[B3] Averill J. R. (1982). Anger and Aggression: An Essay on Emotion. New York, NY: Springer-Verlag.

[B4] Bandura A. (1973). Aggression: A Social Learning Analysis. Englewood Cliffs, NJ: Prentice-Hall.

[B5] Beck A. T. (1999). Prisoners of Hate: The Cognitive Basis of Anger, Hostility, and Violence. New York, NY: HarperCollins Publishers.

[B6] Berkowitz L. (1993). Towards a general theory of anger and emotional aggression: implications of the cognitive-neoassociationistic perspective for the analysis of anger and other emotions, in Perspectives on Anger and Emotion: Advances in Social Cognition, Vol. VI, eds Wyer R. S. Jr. Srull T. K. (Hillsdale, NJ: Lawrence Erlbaum Associates), 1–46.

[B7] Berkowitz L. Harmon-Jones E. (2004). Toward an understanding of the determinants of anger. Emotion 4, 107–130. 10.1037/1528-3542.4.2.107 15222847

[B8] Berrios R. Totterdell P. Kellett S. (2015). Eliciting mixed emotions: a meta-analysis comparing models, types, and measures. Front. Psychol. 6:428. 10.3389/fpsyg.2015.00428 25926805 PMC4397957

[B9] Bettencourt B. Talley A. Benjamin A. J. Valentine J. (2006). Personality and aggressive behavior under provoking and neutral conditions: a meta-analytic review. Psychol. Bull. 132, 751–777. 10.1037/0033-2909.132.5.751 16910753

[B10] Bosch N. D'Mello S. (2014). Co-occurring affective states in automated computer programming education, in Proceedings of the Workshop on AI-supported Education for Computer Science (AIEDCS) at the 12th International Conference on Intelligent Tutoring Systems (Honolulu, HI), 21–30.

[B11] Braniecka A. Trzebinska E. Dowgiert A. Wytykowska A. (2014). Mixed emotions and coping: the benefits of secondary emotions. PLoS ONE 9:e103940. 10.1371/journal.pone.0103940 25084461 PMC4118988

[B12] Costa P. T. Jr McCrae R. R. Dembroski T. M. (1989). Agreeableness vs. antagonism: explication of a potential risk factor for CHD, in In Search of Coronary-Prone Behavior: Beyond Type A, eds Siegman A. Dembroski T. M. (Hillsdale, NJ: Lawrence Erlbaum Associates), 41–63.

[B13] Costa P. T. McCrae R. R. Psychological Assessment Resources I. (1992). Revised NEO Personality Inventory (NEO PI-R) and NEO Five-Factor Inventory (NEO FFI): Professional Manual. Odessa, FL: Psychological Assessment Resources, Inc.

[B14] Dallos R. Vetere A. (2009). Systemic Therapy and Attachment Narratives: Applications in a Range of Clinical Settings. New York, NY: Routledge.

[B15] Davydov D. M. Zech E. Luminet O. (2011). Affective context of sadness and physiological response patterns. J. Psychophysiol. 25, 67–80. 10.1027/0269-8803/a000031

[B16] Dawson J. F. (2014). Moderation in management research: What, why, when and how. J. Bus. Psychol. 29, 1–19. 10.1007/s10869-013-9308-7

[B17] DeWall C. N. Anderson C. A. Bushman B. J. (2011). The general aggression model: theoretical extensions to violence. Psychol. Viol. 1, 245–258. 10.1037/a0023842

[B18] Enders C. K. Tofighi D. (2007). Centering predictor variables in cross-sectional multilevel models: a new look at an old issue. Psychol. Method. 12, 121–138. 10.1037/1082-989X.12.2.121 17563168

[B19] Frijda N. H. (1993). Moods, emotion episodes, and emotions, in Handbook of Emotions, eds. Lewis M. Haviland J. M. (New York, NY: Guilford), 381–403.

[B20] Frijda N. Markam S. Sato K. Wiers R. (1995). Emotions and emotion words, in Everyday Conceptions of Emotion, eds Russel J. A. Fernández-Dols J.-M. Manstead A. S. R. Wellenkamp J. C. (Dordrecht: Kluwer), 121–143.

[B21] Funkenstein D. H. King S. H. Drolette M. (1954). The direction of anger during a laboratory stress-inducing situation. Psychosom. Med. 16, 404–413 13204507 10.1097/00006842-195409000-00006

[B22] Gerstorf D. Siedlecki K. L. Tucker-Drob E. M. Salthouse T. A. (2009). Within-person variability in state anxiety across adulthood: magnitude and associations with between-person characteristics. Int. J. Behav. Dev. 33, 55–64. 10.1177/0165025408098013 24347751 PMC3859617

[B23] Gonzalez R. Smith J. Nielsen L. (2017). Editorial overview: theories, methods, and applications of mixed emotions. Cur. Op. Behav. Sci. 15, 2352–1546. 10.1016/j.cobeha.2017.05.019 29276729 PMC5739087

[B24] Gross J. J. John O. P. (1997). Revealing feelings: facets of emotional expressivity in self-reports peer ratings, and behavior. J. Pers. Soc. Psychol. 72, 435–448. 9107009 10.1037//0022-3514.72.2.435

[B25] Guo Y. Zhang H. Gao J. Wei S. Song C. Sun P.. (2015). Study of genes associated with the ‘anger-in’ and ‘anger-out’ emotions of humans using a rat model. Exp. Ther. Med. 9, 1448–1454. 10.3892/etm.2015.2246 25780450 PMC4353780

[B26] Harley J. Bouchet F. Azevedo R. (2012). Measuring learners' co-occurring emotional responses during their interaction with a pedagogical agent in MetaTutor, in Lecture Notes in Computer Science, Vol. 7315, Intelligent Tutoring Systems, eds Cerri S. A. Clancey W. J. Papadourakis G. Panourgia K. (Berlin; Heidelberg: Springer-Verlag), 40–45.

[B27] Heck R. H. Thomas S. L. Tabata L. N. (2010). Multilevel and Longitudinal Modeling with IBM SPSS. New York, NY: Routledge.

[B28] Hofmans J. Kuppens P. Allik J. (2008). Is short in length short in content? An examination of the domain representation of the ten item personality inventory scales in Dutch Language. Pers. Ind. Diff. 45, 750–755. 10.1016/j.paid.2008.08.004

[B29] Huesmann L. R. (1998). The Role of Social Information Processing and Cognitive Schema in the Acquisition and Maintenance of Habitual Aggressive Behavior, in Human Aggression, eds Russell G. G. Edward D. (San Diego, CA: Academic Press), 73–109.

[B30] Illman N. A. Brown J. S. L. (2016). Reaching out to problem anger: assessing the effectiveness of one-day cognitive behavioural workshops in a community setting in the UK. Behav. Cogn. Psychother. 44, 615–619. 10.1017/S1352465816000126 27086855

[B31] Izard C. E. (1972). Patterns of Emotions: A New Analysis of Anxiety and Depression. San Diego, CA: Academic Press.

[B32] John O. P. Gross J. J. (2004). Healthy and unhealthy emotion regulation: Personality processes, individual differences, and life span development. J. Pers. 72, 1301–1333. 10.1111/j.1467-6494.2004.00298.x 15509284

[B33] Jones S. E. Miller J. D. Lynam D. R. (2011). Personality, antisocial behavior, and aggression: A metaanalytic review. J. Crim. Just. 39, 329–337. 10.1016/j.jcrimjus.2011.03.004

[B34] Kallasmaa T. Allik J. Realo A. McCrae R. R. (2000). The Estonian version of the NEO-PI-R: an examination of universal and culture-specific aspects of the Five-Factor Model. Eur. J. Person. 14, 265–278. 10.1002/1099-0984(200005/06)14:3

[B35] Kashdan T. B. Goodman F. R. Mallard T. M. Dewall C. N. (2016). What triggers anger in everyday life? Links to the intensity, control, and regulation of these emotions, and personality traits. J. Person. 84, 737–749. 10.1111/jopy.12214 26248974

[B36] Kööts L. Realo A. Allik J. (2011). The influence of the weather on affective experience. J. Indiv. Diff. 32, 74–84. 10.1027/1614-0001/a000037

[B37] Kööts L. Realo A. Allik J. (2012). Relationship between linguistic antonyms in momentary and retrospective ratings of happiness and sadness. J. Indiv. Diff. 33, 43–53. 10.1027/1614-0001/a000061

[B38] Kuppens P. Van Mechelen I. Smits D. J. M. De Boeck P. Ceulemans E. (2007). Individual differences in patterns of appraisal and anger experience. Cogn. Emot. 21, 689–713. 10.1080/02699930600859219

[B39] Larsen J. T. Hemenover S. H. Norris C. J. Cacioppo J. T. (2003). Turning adversity to advantage: On the virtues of the coactivation of positive and negative emotions, in A Psychology of Human Strengths: Fundamental Questions and Future Directions for a Posit. Psychol. eds. Aspinwall L. G. Staudinger U. M. (Washington, DC: American Psychological Association, APA), 211–225.

[B40] Larsen J. T. McGraw A. P. Cacioppo J. T. (2001). Can people feel happy and sad at the same time? J. Soc. Person. Psychol. 81, 684–696. 10.1037/0022-3514.81.4.684 11642354

[B41] Lelieveld G.-J. Van Dijk E. Van Beest I. Van Kleef G. A. (2012). Why anger and disappointment affect other's bargaining behavior differently: The moderating role of power and the mediating role of reciprocal and complementary emotions. Pers. Soc. Psychol. Bull. 38, 1209–1221. 10.1177/0146167212446938 22623429

[B42] Lerner J. S. Li Y. Valdesolo P. Kassam K. (2015). Emotion and decision making. Ann. Rev. Psychol. 66, 799–823. 10.1146/annurev-psych-010213-115043 25251484

[B43] Mazerolle P. Piquero A. Capowich G. E. (2003). Examining the links between strain, situational and dispositional anger, and crime: further specifying and testing general strain theory. Youth Soc. 35, 131–157. 10.1177/0044118X03255029

[B44] Mill A. Allik J. Realo A. (2016). Emotional variability predicts tiredness in daily life: an experience sampling study. J. Indiv. Diff. 37, 181–193. 10.1027/1614-0001/a000206

[B45] Mill A. Allik J. Realo A. Valk R. (2009). Age-related differences in emotion recognition ability: a cross-sectional study. Emotion 9, 619–630. 10.1037/a0016562 19803584

[B46] Moeller J. Ivcevic Z. White A. Brackett M. A. (2018). Mixed emotions: Network analyses of intra-individual co-occurrences within and across situations. Emotion. [Epub ahead of print]. 10.1037/emo0000419 29389203

[B47] Mroczek D. Spiro A. Almeida D. (2003). Between- and within-person variation in affect and personality over days and years: How basic and applied approaches can inform one another. Ageing Int. 28, 260–278. 10.1007/s12126-002-1007-z

[B48] Nezlek J. B. (2007). A multilevel framework for understanding relationships among traits, states, situations, and behaviors. Eur. J. Person. 21, 789–810. 10.1002/per.640

[B49] Ngo F. Paternoster R. (2013). Stalking strain, concurrent negative emotions, and legitimate coping strategies: a preliminary test of gendered strain theory. Am. J. Crim. Just. 38, 369–391. 10.1007/s12103-012-9179-x

[B50] Pease C. R. Lewis G. J. (2015). Personality links to anger: evidence for trait interaction and differentiation across expression style. Pers. Indiv. Diff. 74, 159–164. 10.1016/j.paid.2014.10.018

[B51] Pond R. S. Jr. Kashdan T. B. DeWall C. N. Savostyanova A. A. Lambert N. M. Fincham F. D. (2012). Emotion differentiation moderates aggressive tendencies in angry people: a daily diary analysis. Emotion 12, 326–337. 10.1037/a0025762 22023359

[B52] Reinard J. (2006). Communication Research Statistics. Thousand Oaks, CA: Sage.

[B53] Reisenzein R. (2009). Emotional experience in the computational belief-desire theory of emotion. Emotion Rev. 1, 214–222. 10.1177/1754073909103589

[B54] Russell J. A. (2003). Core affect and the psychological construction of emotion. Psychol. Rev. 110, 145–172. 10.1037/0033-295X.110.1.145 12529060

[B55] Sanford K. (2012). The communication of emotion during conflict in married couples. J. Fam. Psychol. 26, 297–307. 10.1037/a0028139 22545937

[B56] Scherer K. R. (2013). Measuring the meaning of emotion words: a domain-specific componential approach, in Components of Emotional Meaning: A Sourcebook, eds Fontaine J. R. J. Soriano K. R. S. C. (Oxford: Oxford University Press).

[B57] Skarlicki D. P. Folger R. (1997). Retaliation in the workplace: the roles of distributive, procedural, and interactional justice. J. Appl. Psychol. 82, 434–443.

[B58] Spielberger C. D. (2010). State-Trait Anxiety Inventory. in The Corsini Encyclopedia of Psychology, 4th edn., eds Weiner I. B. Craighead W. E. (Hoboken, NJ: Wiley), 1698–1701. 10.1002/9780470479216.corpsy0943

[B59] Spielberger C. D. Johnson E. H. Russell S. F. Crane R. J. Jacobs G. A. Worden T. I. (1985). The experience and expression of anger: construction and validation of an anger expression scale, in Anger and Hostility in Cardiovascular and Behavioral Disorders, eds Chesney M. A. Rosenman R. H. (New York, NY: Hemisphere/McGraw-Hill), 5–30.

[B60] Thompson R. J. Mata J. Jaeggi S. M. Buschkuehl M. Jonides J. Gotlib I. H. (2011). Concurrent and prospective relations between attention to emotion and affect intensity: an experience sampling study. Emotion 11, 1489–1494. 10.1037/a0022822 21534663 PMC3204007

[B61] Trampe D. Quoidbach J. Taquet M. (2015). Emotions in everyday life. PLoS ONE 10:e0145450. 10.1371/journal.pone.0145450 26698124 PMC4689475

[B62] Tugade M. M. Fredrickson B. L. (2004). Resilient individuals use positive emotions to bounce back from negative emotional experiences. J. Pers. Soc. Psychol. 86, 320–333. 10.1037/0022-3514.86.2.320 14769087 PMC3132556

[B63] Van Dijk W. W. Van Harreveld F. (2008). Disappointment and regret,. in *Research Companion to Emotions in Organizations*, eds Ashkanasy N. M. Cooper C. L. (London: Edward Elgar Publishers), 90–102.

[B64] Van Kleef G. A. De Dreu C. K. W. Manstead A. S. R. (2004). The interpersonal effects of anger and happiness in negotiations. J. Pers. Soc. Psychol. 86, 57–76. 10.1037/0022-3514.86.1.57 14717628

[B65] Watson D. (2000). Emotions and Social Behavior. Mood and Temperament. New York, NY: Guilford Press.

[B66] Wilkowski B. M. Robinson M. D. (2008). Clear heads are cool heads: emotional clarity and the down-regulation of antisocial affect. Cogn. Emot. 22, 308–326. 10.1080/02699930701394199

[B67] Wilkowski B. M. Robinson M. D. (2010). The anatomy of anger: an integrative cognitive model of trait anger and reactive aggression. J. Person. 78, 9–38. 10.1111/j.1467-6494.2009.00607.x 20433611

[B68] Wubben M. J. J. De Cremer D. Van Dijk E. (2011). The communication of anger and disappointment helps to establish cooperation through indirect reciprocity. J. Econ. Psychol. 32, 489–501. 10.1016/j.joep.2011.03.016

[B69] Zelenski J. M. Larsen R. J. (2000). The distribution of basic emotions in everyday life: a state and trait perspective from experience sampling data. J. Res. Personality 34, 178–197. 10.1006/jrpe.1999.2275

[B70] Zhan J. Ren J. Fan J. Luo J. (2015). Distinctive effects of fear and sadness induction on anger and aggressive behavior. Front. Psychol. 6:725. 10.3389/fpsyg.2015.00725 26124725 PMC4467173

