# Supplementary material for: The Role of Co-occurring Emotions and Personality Traits in Anger Expression
Source: Front Psychol. 2018 Feb 9;9:123. doi: 10.3389/fpsyg.2018.00123 (PMC5811638; doi:10.3389/fpsyg.2018.00123)
Supplement: Supplementary file 1 [file Table1.PDF]

Appendix 1. Means and standard deviations of predictor and outcome variables

Variable	*M*	*SD*
Anger	2.35	0.61
Fear	1.32	0.62
Sadness	1.89	0.91
Surprise	1.55	0.83
Disgust	1.52	0.83
Contempt	1.11	0.38
Disappointment	2.09	0.97
Irritation	2.23	0.92
Neuroticism	84.36	29.78
Extraversion	103.73	29.71
Openness to Experience	104.23	31.10
Agreeableness	119.34	27.44
Conscientiousness	114.32	30.22
Age	39.88	23.29
Anger-in	2.82	0.76
Anger-out	1.92	0.63

N = 110
